# Evaluation of EGFR, KRAS and BRAF gene mutations in renal cell carcinoma

**DOI:** 10.15586/jkcvhl.2014.10

**Published:** 2014-08-05

**Authors:** Omer Bayrak, Haluk Sen, Ersan Bulut, Beyhan Cengiz, Metin Karakok, Sakip Erturhan, Ilker Seckiner

**Affiliations:** 1 Department of Urology, 3 Department of Physiology, 4 Department of Pathology, University of Gaziantep, Gaziantep, Turkey; 2 Department of Medical Genetics, University of Gazi, Ankara, Turkey

## Abstract

A subset of renal cell carcinoma (RCC) patients has been shown to respond to anti-EGFR therapy. As KRAS and BRAF mutations are associated with poor response to anti-EGFR therapy in some cancers, it has been suggested that screening for KRAS and BRAF mutations in RCC may be a promising strategy to identify patients who might respond to EGFR-targeted therapy. The aim of this study was to investigate the mutation status of EGFR, KRAS and BRAF in RCC patients. Renal tumors and normal renal samples from forty-eight patients who underwent radical or partial nephrectomy for kidney cancer were used in this study. Histological classification of the tumors was performed according to International Union against Cancer (UICC) / American Joint Committee on Cancer (AJCC) classification. Seventeen patients (48%) had clear-cell RCC, 7 (20%) had chromophobe RCC, and 11 patients (32%) had papillary RCC. DNA isolated from the samples was subjected to melting curve mutation analysis for EGFR, BRAF and KRAS using ABI-3130 DNA sequencer. DNA sequencing analysis of RCC samples, when compared with morphologically normal matched regions, did not show any exon mutations. Our results do not support the notion that EGFR, KRAS and BRAF might be mutated in RCC.

## INTRODUCTION

Renal cell carcinoma (RCC) constitutes 3% of all adult malignancies (1). According to Surveillance, Epidemiology, and End Results (SEER) data, the annual increase in RCC incidence is 2.5–3%, as we have started to use modern imaging methods more frequently since 1970s (2). Although 60% of new diagnoses are coincidental, 25% of the patients are metastatic during the diagnosis (3). Radical nephrectomy or nephron sparing surgery is the standard treatment for localized RCC, while 30% of the patients experience recurrence after the surgery (3). Despite the tremendous improvements in our understanding of the molecular mechanisms of RCC, and the introduction of many novel multi-tyrosine kinase inhibitors in clinical practice for the treatment of RCC the five-year survival of metastatic patients continues to be less than 10%. There is a need for a better understanding of the molecular mechanisms of RCC and the discovery of more efficient therapeutics for the management of metastatic RCC. The epidermal growth factor receptor (EGFR), a transmembrane tyrosine kinase receptor of the Erb family, is overexpressed in both primary and metastatic RCC (4–6) suggesting the potential of anti-EGFR agents as therapeutics for the treatment of RCC. While anti-EGFR therapy demonstrated effective anti-tumoric activity in laboratory settings (7, 8), clinical trials demonstrated a very low objective response (9). Of the 88 patients treated with ABX-EGF, one complete, two partial, and two minor responses were observed (9). While the reasons for these disappointing results are not clear, it is possible that mutations of KRAS and BRAF are involved. This notion stems from the fact that, in colorectal cancer, mutations of KRAS/BRAF genes, which are integral part of the EGFR signaling pathway make EGFR inhibitors ineffective (10, 11). On the contrary, a case report demonstrates that EGFR mutations could sensitize patients to anti-EGFR therapy (12). Therefore, screening for EGFR, KRAS and BRAF mutations in RCC may be a promising strategy to identify patients who might respond to EGFR-targeted therapy. The present study aims to identify EGFR, KRAS and BRAF mutations in RCC.

## MATERIALS AND METHODS

### Patient Selection

After obtaining local ethics committee approval, RCC and matched normal samples from 48 patients who underwent radical or partial nephrectomy for kidney cancer were evaluated between June 2009 and June 2011 at the University of Gaziantep, Department of Urology, Turkey. Thirteen patients who had benign and ureteral carcinoma according to the pathological results were excluded from the study. The samples from the remaining 35 patients were used for further study. Portion of the samples were formalin-fixed and processed for histology and the remaining were stored at -80^o^C until use.

### Histology

Three micron sections of the formalin-fixed kidney samples were stained with hematoxyline and eosine and the tumor grade was determined according to International Union against Cancer (UICC) / American Joint Committee on Cancer (AJCC) 2009 TNM classification, whereas tumor nuclear grading was performed according to the Fuhrman grading system by a qualified Pathologist.

### Mutation Detection

DNA from kidney samples that had been stored at -80^o^C (30–50 mg tissue) were isolated using Roche High Pure Polymerase Chain Reaction (PCR) Template Preparation Kit (Catalogue Number: 11 796 828 001) following the protocol of the supplier. DNA samples were stored at -20°C until further use. DNA sequencing was performed on an ABI 3130 DNA sequencing analysis instrument. The target area was amplified by PCR using primers specific to EGFR, KRAS, and BRAF (Table 1).

**Table 1.** PCR primers and product lengths of EGFR, BARF and KRAS

Gene	Exon		Primer sequences	Product length (bp)
**EGFR**	18	Forward	GTGAGGGCTGAGGTGACC	186
		Reverse	TGTGCCAGGGACCTTACC	
	19	Forward	TGCCAGTTAACGTCTTCC	155
		Reverse	CACAGCAAAGCAGAAACTC	
	21	Forward	TCTTCCCATGATGATCTGTC	225
		Reverse	GACCTAAAGCCACCTCCT	
**BRAF**	11	Forward	TGTTTGGCTTGACTTGAC	176
		Reverse	CACCACATTACATACTTACC	
	15	Forward	TACTGTTTTCCTTTACTTAC	165
		Reverse	TAGCCTCAATTCTTACCA	
**KRAS**	1	Forward	GGCCTGCTGAAAATGACTGA	162
		Reverse	GTCCTGCACCAGTAATATGC	
	2	Forward	CTGTAATAATCCAGACTGTG	151
		Reverse	TCCCCAGTCCTCATGTACTG	

The primers were designed specifically for the most mutation presenting regions of EGFR, BRAF and KRAS genes. These regions contained exons 18, 19, 21 for EGFR, exons 11, 15 for BRAF and exons 1, 2 for KRAS genes (13–15). The PCR conditions were the same for all PCR reactions. PCR products were visualized with agarose gel electrophoresis. After detecting the optimal size of PCR product, DNA sequencing was performed using BigDye® Terminator v1.1 Cycle Sequencing Kit (Applied Biosystem, SKU#4337450).

The PCR mixture was kept at 96°C for 1 minute. Then, PCR was carried out with 25 cycles consisting of following steps: 10 seconds at 96°C, 5 seconds at 50°C, and 4 minutes at 60°C. Samples were kept at 4°C until they were placed in the instrument. In automated DNA sequencing, PCR products were loaded into the instrument after a clean-up step through Sephadex. To do this, 1 g of Sephadex was dissolved in 14 ml of ultrapure water, and 600 μl of this solution was transferred to the columns. After centrifugation at 2000xg for 2 minutes, Sephadex-containing columns were transferred to other tubes, and 10 μl of PCR product was added on Sephadex. Centrifugation was performed at 2000xg for 2 minutes. Following centrifugation, the products at the bottom of the tube were subjected to DNA sequencing by Sanger dye-terminator sequencing method. Each dideoxynucleotide in the DNA sequence analysis was labelled with a different fluorescence dye. Amplified DNA fragments were migrated through a “gel matrix”, which were loaded in capillaries, and detected by an instrument capable of recognizing fluorescent dyes.

## RESULTS

Nineteen male and 16 female patients (35 patients in total) who had RCC were included in the study. The mean age of the patients was calculated as 59.31 ± 12.52 (15–77) years. None of the patients were in an occupational group that might play a role in kidney cancer etiology. History of smoking was present in ten male patients (52.6%) and in four female patients (25%). The mean body mass index was 28.31 ± 3.45 (21–33) kg/m^2^. According to histopathological UICC and AJCC classification systems, 17 patients (48%) had clear-cell RCC, 7 patients (20%) had chromophobe cell RCC, and 11 patients (32%) had papillary RCC. According to 2009 TNM staging of the tumors, 11 patients (31%) were T1a, 8 patients (23%) were T1b, 3 patients (8%) were T2, 9 patients (26%) T3a, and 4 patients (12%) T3b. Twenty-three patients (65%) were evaluated as N0, 8 were (23%) N1, and 4 patients were (12%) N2. According to the Fuhrman grading system, 3 patients (8%) were Grade 1, 15 patients (43%) were Grade 2, and 17 patients were (49%) Grade 3 (Table 2). DNA sequencing analysis of cancer samples and normal tissues did not show any exon mutations in the EGFR, BRAS, and KRAS pathway (data not shown).

**Table:** **Table 2.** Characteristics of patient samples

RCC subtype	%	Number of samples
Clearcell RCC	48%	(17/35)
Papillary RCC	32%	(11/35)
Chromophobe RCC	20%	(7/35)
TNM		
T1a	31%	(11/35)
T1b	23%	(8/35)
T2	8%	(3/35)
T3a	26%	(9/35)
T3b	12%	(4/35)
N0	65%	(23/35)
N1	23%	(8/35)
N2	12%	(4/35)
Fuhrman’s Classification		
Grade 1	8%	(3/35)
Grade 2	43%	(15/35)
Grade 3	49%	(17/35)

## DISCUSSION

BRAF and KRAS belong to the RAF proto-oncogene serine / threonine-protein kinase (c-RAF) gene family and their over expression or mutations trigger abnormal cell proliferation. EGFR is believed to be responsible for cell proliferation during carcinogenesis (16). Kamai et al. (17) evaluated the association of parathyroid hormone-related protein (PTHrP) and KRAS in RCC. Of the 51 patients, serum PTHrP and mRNA expression of KRAS were significantly high in 7 patients (17). Also, there was a correlation between high KRAS expression and PTHrP-induced hypercalcemia. However, the mutation status of KRAS was not studied. Kozma et al. (18) analyzed 36 RCC samples for c-myc and KRAS amplification. Three samples (8.3%) showed c-myc, and 6 samples (16.6%) displayed KRAS amplifications. The authors also reported that the amplifications correlated with tumor grade and size but not with lymph node involvement. In a comprehensive analysis of 121 RCC samples, KRAS and BRAF did not reveal any mutations (19). In a multicenter study, Szymanska et al. (20) investigated the correlation between TP53 (exons 5–9), EGFR (exons 18–21) and KRAS (codon 12) mutation and Von Hippel-Lindau (VHL) gene in tissue samples derived from 361 RCC (334 clear-cell carcinomas) patients. The authors observed TP53 mutation in 4% of clear-cell carcinoma subtypes, which was independent of VHL mutations. EGFR and KRAS mutations were not detected in any patients. The authors concluded that TP53, KRAS, or EGFR mutations do not have a major contribution to RCC development, provided that the VHL gene is not inactivated (20). Furthermore, Sakaeda and colleagues reported no mutations of EGFR in a cohort of Japanese patients (21). We studied EGFR, BRAF and KRAS mutation in a Turkish cohort, and did not find any mutations, corroborating previous findings. Screening for EGFR, KRAS and BRAF mutations in RCC is unlikely to be a promising strategy to identify patients who might respond to EGFR-targeted therapy.
